# Macrodissection versus microdissection of rectal carcinoma: minor influence of stroma cells to tumor cell gene expression profiles

**DOI:** 10.1186/1471-2164-6-142

**Published:** 2005-10-14

**Authors:** Elza C de Bruin, Simone van de Pas, Esther H Lips, Ronald van Eijk, Minke MC van der Zee, Marcel Lombaerts, Tom van Wezel, Corrie AM Marijnen, J Han JM van Krieken, Jan Paul Medema, Cornelis JH van de Velde, Paul HC Eilers, Lucy TC Peltenburg

**Affiliations:** 1 Department of Clinical Oncology, Leiden University Medical Center, Albinusdreef 2, 2333 ZA Leiden, The Netherlands; 2 Department of Pathology, Leiden University Medical Center, Albinusdreef 2, 2333 ZA Leiden, The Netherlands; 3 Department of Pathology, University Medical Center St. Radboud, Geert Grooteplein-Zuid 10, 6525 GA Nijmegen, The Netherlands; 4 Department of Surgery, Leiden University Medical Center, Albinusdreef 2, 2333 ZA Leiden, The Netherlands; 5 Department of Medical Statistics, Leiden University Medical Center, Wassenaarseweg 62, 2333 AL, Leiden, The Netherlands

## Abstract

**Background:**

The molecular determinants of carcinogenesis, tumor progression and patient prognosis can be deduced from simultaneous comparison of thousands of genes by microarray analysis. However, the presence of stroma cells in surgically excised carcinoma tissues might obscure the tumor cell-specific gene expression profiles of these samples. To circumvent this complication, laser microdissection can be performed to separate tumor epithelium from the surrounding stroma and healthy tissue. In this report, we compared RNAs isolated from macrodissected, of which only surrounding healthy tissue had been removed, and microdissected rectal carcinoma samples by microarray analysis in order to determine the most reliable approach to detect the expression of tumor cell-derived genes by microarray analysis.

**Results:**

As microdissection yielded low tissue and RNA quantities, extra rounds of mRNA amplification were necessary to obtain sufficient RNA for microarray experiments. These second rounds of amplification influenced the gene expression profiles. Moreover, the presence of stroma cells in macrodissected samples had a minor contribution to the tumor cell gene expression profiles, which can be explained by the observation that more RNA is extracted from tumor epithelial cells than from stroma.

**Conclusion:**

These data demonstrate that the more convenient procedure of macrodissection can be adequately used and yields reliable data regarding the identification of tumor cell-specific gene expression profiles.

## Background

Microarray technology permits simultaneous analyses of the expression profiles of thousands of genes. These analyses allow identification of profiles correlating with prognosis and permit tumor classifications [1-3], but can also be used to identify genes that are involved in several molecular processes, like carcinogenesis, metastasis and responses to treatment (reviewed by ref [4]).

To ensure that the expressions of tumor cell-derived genes are identified by microarray analysis of surgically excised carcinomas, the samples can be enriched for tumor cells by removing the surrounding healthy tissue. However, besides tumor epithelium with infiltrating cells, these macrodissected samples contain stroma cells as well. Evidently, after RNA isolation of such macrodissected samples, tumor epithelium-derived RNA cannot be separated from RNA specific for stroma. Although informative, the presence of stroma might obscure the tumor cell gene expressions, thereby preventing accurate data on tumor cell expression profiles. Because in rectal carcinoma the percentages of stroma versus tumor epithelium vary widely among patients, this high variation might complicate comparisons of different tumor samples even more.

To circumvent this problem, microdissection, such as Laser Microdissection and Pressure Catapulting (LMPC), can be used to select tumor epithelial cells exclusively. Although contamination of infiltrating cells will in this case also be present and important micro environmental information of the tumor cells will be missed, RNA extracted from such microdissected samples is expected to be more specific for tumor epithelial gene expression than RNA isolated from macrodissected samples. Comparisons of gene expression profiles of a small number of carcinoma samples obtained using macrodissection or microdissection, indeed led to the conclusion that stroma cells disturb the tumor gene expression profiles [5]. However, it has also been demonstrated that some degradation of RNA occurs during the lengthy procedure of laser capture microdissection, resulting in a decreased correlation between macro- and randomly microdissected samples [6]. Another disadvantage of microdissection can be the limited amount of extracted RNA, requiring an extra amplification round to get sufficient RNA for microarray experiments. There are several publications addressing the effect of amplification on gene expression profiles. T7 polymerase-based mRNA amplification is demonstrated to be reproducible and to maintain the relative abundances of mRNA transcripts, although lower correlation coefficients are always observed when amplified samples are compared to non-amplified samples [7-9]. This amplification effect becomes more serious with less starting material, which is the case for microdissected samples. In addition, a second round of amplification does have a further effect on reproducibility [10,11].

In this study, we determined the most reliable way to detect the expression of tumor cell-derived genes by microarray analysis: macrodissection or microdissection. Comparing gene expression profiles of macrodissected and microdissected rectal carcinoma samples in the same experimental setting allowed evaluation of the effect of a second round of RNA amplification as well as evaluation of the presence of varying amounts of stroma. Quantification of both effects demonstrated that the second amplification round had a high impact on gene expression profiles. In addition, epithelial tumor cells as compared to stroma cells had a much higher contribution to gene expression profiles than is expected from the quantified surface percentage. We conclude that the obscuring effect of stroma on the tumor epithelium gene expression profiles appears to be minimal and that therefore in clinical settings the convenient procedure of macrodissection is the preferable method to examine rectal carcinomas by microarray analysis.

## Results

### Gene expression profiles of macrodissected and microdissected rectal carcinomas

In the panel of excised rectal carcinoma samples used for this study, a high variation in surface percentages of tumor epithelium versus stroma was observed; percentages of epithelium ranged from 11 to 82% (Table 1). In order to compare macro- and microdissection of these carcinoma samples in microarray experiments, RNA was extracted from carcinoma tissue where surrounding healthy tissue had been removed (macrodissection), as well as from tumor epithelium isolated by LMPC (microdissection) of the same carcinoma samples. The microdissection procedure of tumor epithelium resulted on average in 30 ng of total RNA. Because 1 μg of mRNA is normally required for microarray experiments, two rounds of mRNA amplification were necessary, yielding on average 15 μg aRNA. For macrodissected samples one round of mRNA amplification was sufficient to get an adequate amount of aRNA. To be able to examine the effect of the second round of mRNA amplification on the gene expression profiles, several macrodissected samples were amplified a second round as well (Table 1).

**Table 1:** Percentages epithelial tumor surface and used amplification scheme

sample	% tumor	amplification rounds		
		
	epithelium	macro	micro	
			
			tumor	stroma
1	11	1	2	nd
2	15	1, 2	2	2
3	17	1	2	2*
4	21	1	2	nd
5	35	1, 2	2	nd
6	37	1	2	nd
7	43	1	2	nd
8	50	1, 2	2	2
9	52	1	2	nd
10	59	1, 2	2	nd
11	59	1, 2	2	2
12	61	1	2	nd
13	71	1	2	nd
14	82	1, 2	2	2

* sample not used for microarray experiment because of insufficient aRNA after amplification.

**Figure 1:**
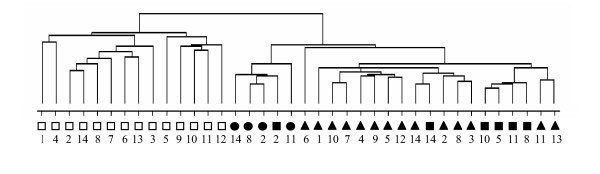
Unsupervised hierarchical clustering of macro- and microdissected rectal carcinoma samples. Macrodissected samples (squares), microdissected tumor epithelium samples (triangles) and microdissected stroma samples (circles) were clustered based on average correlation. Open symbols indicate RNA analyzed after one round of amplification and closed symbols indicate two rounds of amplification. Numbers correspond to the carcinoma samples in Table 1.

All samples were Cy5-labeled and mixed with an equal amount of Cy3-labeled reference probe, consisting of equal amounts of RNA of all macrodissected samples. After hybridization of cDNA arrays, data were normalized and filtered, resulting in a set of 2358 genes that gave sufficient signal on all arrays. Based on the expression of these 2358 genes, hierarchical clustering was performed to group samples according to similarity in gene expressions, without information of sample identity (Figure 1). This unsupervised clustering distinguished two main clusters according to the number of amplification cycles. Macrodissected samples that were amplified a second round were more similar to twice-amplified microdissected samples than to their original once-amplified samples. To determine the statistical significance of the effect of the second amplification cycle, Pearson correlation coefficients between once-amplified macrodissected samples and their corresponding twice-amplified samples were calculated. The resulting coefficients were low, in contrast to the coefficients of independently amplified samples or of duplicate labeling experiments (Table 2), excluding that such a change in expression profiles was induced by experimental variation.

**Table 2:** Effect of the second round of amplification. Pearson correlation coefficients evaluating the effect of the second round of amplification on the gene expression profiles. Twice-amplified macrodissected samples were compared to the corresponding once-amplified macrodissected samples. Correlation of duplicate amplification and labeling experiments are presented as well. In case of repeated experiments, Pearson correlation coefficients were calculated for each experiment and averaged.

Sample	Correlation Coefficient	p-value
2	0.24	<0.001
5	0.24	<0.001
8	0.00	0.961
10	0.19	<0.001
11	0.17	<0.001
14	0.15	<0.001

labelling	0.95	<0.001
amplification	0.81	<0.001

Taken together, these findings indicate that, in this experimental setting, expression profiles were hardly preserved during the extra round of amplification performed with random primers, and therefore exclude reliable cross-comparison of once- and twice-amplified samples.

### Evaluation of the bias introduced by second round amplification

Low correlation coefficients indicate that the overall gene expression profile was changed. However, such coefficients do not specify whether the expression of all genes was slightly changed or whether the expression of a proportion of genes was altered dramatically. To evaluate the amplification-induced change in more detail, the number of genes that were significantly preserved by the second round of amplification, a "conservative set of genes", was defined by t-tests according to Nygaard et al. [9]. These calculations indicated that 42% of the genes were on average not significantly influenced. Calculating a "rejected set of genes" indicated that for 20% of the genes the expressions were significantly changed, and most of these rejected genes (70%) were changed at least three-fold. This suggests that a substantial proportion of the expression profile was significantly affected by the second round of amplification with random primers. However, closer examination of this "rejected gene-set" revealed that for the majority of the rejected genes, the amplification-induced change was in the same direction over all tumor samples, indicating that the bias for these genes could be constant. Although changed significantly, a constant bias might not influence the outcome as long as all tested samples are amplified for the same number of cycles.

**Figure 2:**
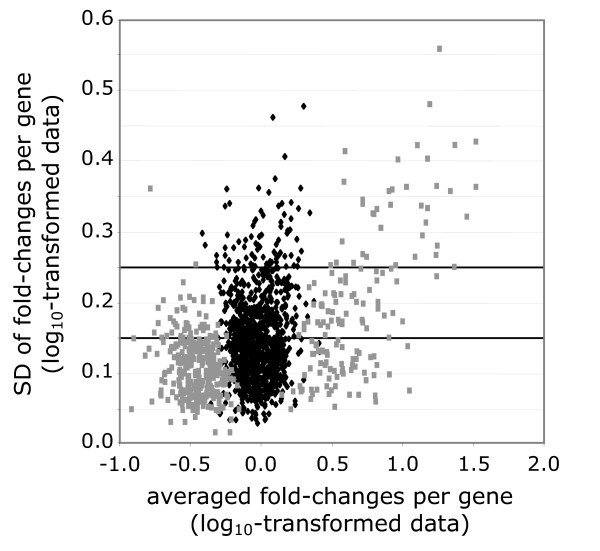
Variation in amplification-induced fold-change for the conserved and rejected gene-sets. Per gene the fold-change induced by the second round of amplification over all samples was averaged (x-axis) and plotted against the standard deviation (SD) of the fold-change (y-axis). Statistically conserved genes (black) and rejected genes (gray) displayed high variation in standard deviations. A cut-off value at which the 95% normal CI lies between 0.5 and 2 times the expression value (± 0.3 on log_10_-scale) corresponds to an SD of 0.15 (the 95% normal CI lies within 1.96 standard deviations of the mean; in this case SD = 0.3/1.96 = 0.15).

To analyze whether the amplification-induced bias was constant for all carcinoma samples, we determined the actual variation for the whole set of 2358 genes. This variation between once- and twice-amplified macrodissected samples is more indicative for the reproducibility of the amplification effect on the gene expression profiles. Therefore, the amplification-induced fold-changes of each gene were calculated for all tumor samples. These values were then averaged, which allow calculation of a standard deviation (SD) of the fold-change for each gene (Figure 2). A high SD indicates that the amplification-induced change of that gene was less reproducible over the different samples. For instance, the expression of a given gene may be induced ten-fold in one sample, while reduced ten-fold in the next sample. On average, the amplification-induced change is zero, suggesting no effect. However, the variation of this particular gene among samples is 100-fold, which is too high to be regarded as reproducible. To determine which variation-range is acceptable, we used the 95% normal confidence interval, which is defined by the mean ± 1.96*SD. An interval with a ten-fold variation-range has an SD of 0.25 on a log_10_-based scale, and an interval with a four-fold variation-range has an SD value of 0.15. Figure 2 demonstrates that several genes (8%) have a higher SD than 0.25, indicating that for these genes the amplification resulted in a highly dispersed (>ten-fold) expression pattern. When the cut-off point of the SD was set at 0.15, a range we propose to be acceptable, it turned out that 39% of all genes had a higher standard deviation. For this substantial proportion of genes, we concluded that the amplification effect was not constant over the different samples.

### Larger contribution of tumor epithelium than stroma to gene expression profiles

**Figure 3:**
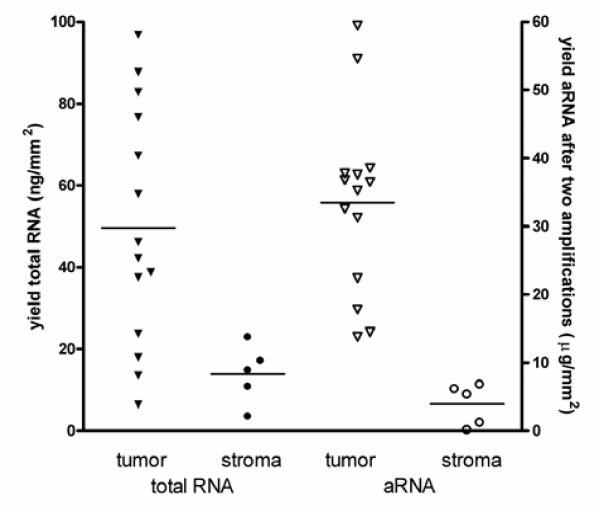
Total RNA and amplified RNA yields of equal volumes of microdissected tumor epithelium and stroma. The average yield of total RNA (left axis; closed symbols) isolated from tumor epithelium (triangles; mean 50 ng/mm^2 ^of 10 μm thick sections), was higher than total RNA isolated from stroma (circles; mean 14 ng/mm^2^; p = 0.001). After two rounds of amplification, a higher difference was observed between yields of RNA (right axis; open symbols) of microdissected tumor epithelium (mean 33 μg/mm^2^) and stroma (mean 4 μg/mm^2^; p < 0.0001).

In the unsupervised clustering (Figure 3) all twice-amplified samples clustered together. In this subgroup the macrodissected samples clustered closer to microdissected tumor samples than to stroma samples. This observation suggested that in general the effect of stroma on gene expression profiles of macrodissected samples was smaller than the contribution of tumor epithelium. To determine the contributions of epithelial tumor cells and of stroma cells in the gene expression profiles of macrodissected samples, linear regression analysis was performed on the twice-amplified macrodissected samples with their corresponding microdissected tumor and stroma samples (Table 3). In this analysis, the relationship between the macrodissected sample and the corresponding tumor and stroma samples were quantified according to the formula: gene expressions of macrodissected sample = α*tumor expressions + β*stroma expressions. The relative contribution of tumor epithelium can then be calculated by α/(α+β). In case of carcinoma sample 14, with 18% stroma and 82% tumor epithelial surface in the macrodissected section, the relative contributions of stroma and tumor mRNA were 7% and 93%, respectively. For carcinoma sample 2, which had only 15% tumor epithelium surface in the macrodissected section, stroma and tumor mRNAs contributed equally to the gene expression profile of the macrodissected sample. These linear regression analyses demonstrate that the macrodissected gene expression profile depends much more on the tumor epithelium than would be expected from the percentage of epithelial tumor surface.

**Table 3:** Involvement of tumor epithelium and stroma. Linear regression was used to quantify the relative contributions of tumor epithelium and stroma to the gene expression profile of the macrodissected sample. If, for one gene, *s *is the amount of RNA measured in microdissected stroma, *t *is the amount measured in microdissected tumor, and *r *is the amount RNA measured in the macrodissected sample, we assume *r *= *αt*+*βs*, where *α *and *β *are unknown coefficients. The last column is the relative contribution of tumor epithelium, α/(α+β), assuming that the contributions of stroma and tumor together are 100%. Because we are considering a sum of contributions on the linear RNA scale, the regression has to be performed on the non-logged data. The values are averaged in case of duplicate labeling experiments and standard errors of coefficients α and β are given.

Sample	surface % epithelium	tumor α (std error)	stroma β (std error)	relative tumor contribution: α/(α+β)
2	15	0.44 (0.01)	0.46 (0.01)	49%
5	35	0.80 (0.01)	nd	nd
8	50	0.93 (0.01)	0.06 (0.02)	94%
10	59	0.91 (0.01)	nd	nd
11	59	0.66 (0.01)	0.14 (0.02)	83%
14	82	0.98 (0.02)	0.07 (0.03)	93%

The relatively high contribution of epithelial tumor cells suggested that more RNA could be extracted from tumor epithelium than from stroma. Therefore, the yields of total RNA isolated per volume microdissected tissue were compared (Figure 3). Although similar volumes of tumor epithelium and stroma were microdissected, yields of total RNA of epithelial tumor samples were on average 3.5-fold higher than yields of stroma RNA (p = 0.001). This difference between tumor epithelium and stroma increased when the aRNA yields after two rounds of amplification were compared. On average, the amount of aRNA generated from microdissected tumor samples was eight times higher than the aRNA of equal volumes of stroma samples (p < 0.001). This difference in mRNA quantities explained the minor contribution of stroma to the gene expression profiles of macrodissected samples.

## Discussion

Surgically resected rectal carcinomas contain epithelial tumor cells as well as stroma cells. In microarray experiments of such specimens, both components will contribute to the gene expression profiles. The influence of stroma cells might therefore prevent accurate analysis of gene expressions specific for epithelial tumor cells, especially when high percentages of stroma are present in the carcinoma samples. For rectal carcinomas, the observed high variation in percentages of epithelial tumor surface might complicate interpretations of microarray data even more. Therefore, the question arose whether these samples had to be microdissected to obtain reliable tumor epithelial gene expression data.

In this study, we compared gene expression profiles of several macrodissected rectal carcinoma samples, where only surrounding healthy tissue was removed, with the same samples microdissected by LMPC. Both the effect of a second amplification round as well as the effect of stroma on the gene expression profiles was analyzed in order to determine the best dissection method to detect the expression of epithelial tumor-derived genes by microarray analysis. Unsupervised clustering of the gene expression profiles resulted in two main clusters according to the number of amplification rounds. This observation indicates that the second round of amplification, needed for microdissected samples to get sufficient RNA for microarray experiments, affected the overall gene expression profiles.

The T7 RNA polymerase-based linear amplification protocol [12] is one of the most widely used among the available amplification techniques. In this procedure, the amplification reaction consists of transcription via an oligo(dT)-primer harboring a T7 promoter sequence. When second amplification rounds were required, as is the case for microdissected samples, an additional cDNA synthesis step was performed with second round primers followed by the T7-based amplification reaction. Because these second round primers are random primers, transcript sizes will decrease. Quality analysis of the amplified RNA samples demonstrated that indeed the second round of amplification slightly reduced transcript fragments (data not shown). This effect was more pronounced for microdissected samples, probably because of lower amounts of input RNA for the amplification procedure and some degradation occurring during the time-consuming process of microdissection [6].

Most studies determined the amplification effect by comparing expression ratios of two non-amplified RNA samples versus the ratios of the same RNAs amplified. These studies show that the majority of expression differences were maintained by the amplification procedure although a slight decrease in correlation coefficients was observed [13,14], and the intensity levels were not preserved [7,9,15]. In order to evaluate macro- versus microdissection, we determined the effect of the for microdissection required second amplification reaction on the gene expression profiles by comparing once- and twice-amplified samples. The low Pearson correlation coefficients and the calculated significantly "conserved" and "rejected" gene-sets according to Nygaard et al. [9] demonstrate that the overall gene expression profile was changed by the second round of amplification. In this cross-comparison analysis, the extreme low correlation coefficients might be the consequence of the above-suggested loss of intensity levels.

Such a cross-comparison analysis of once- and twice-amplified samples indicates that the gene expression profile is changed by the amplification reaction, but not whether this change is reproducible for all samples. Other studies established that amplification-induced changes are particularly sequence dependent and not abundance dependent [10,15], suggesting a fairly constant bias. Therefore, the variation of the amplification-induced change over the different samples was determined, as this variation will be indicative for the consistency of the bias. When we take a standard deviation of 0.15 as an approximate quantitative criterion (95% normal confidence interval, allowing a four-fold variation in expressions induced by amplification), for 39% of the genes the variation in the gene expression introduced by amplification was outside this confidence interval. This analysis indicates that for a substantial proportion of the genes, the amplification-induced change was not constant.

Importantly, such a high variation was observed with similar frequencies in the "conserved" and "rejected" gene-sets. Therefore, although twice-amplified genes might be called "conserved" based on a t-test, indicating that the change on average is around zero, the variation over the different samples will be changed by the amplification, resulting in more false-negative and false-positive genes. Since genes extracted from the microarray analysis require verification by other biochemical experiments, false-positive genes will be recognized and can be reclassified. Putative interesting genes that are false negative will be missed from the analysis.

Since the second round of amplification affected the gene expression profiles, the use of once-amplified samples is highly preferred. The fact that for macrodissected samples one round of amplification suffices to get enough labeled mRNA, which results in a far more convenient and cost-effective procedure [16], supports the use of macrodissected samples. Although the first amplification round might induce some changes in gene expression as well, the amplification-induced bias is reported to be larger when the amounts of input material is low [10,17]. The yields of RNA isolated from microdissected samples were small, while for macrodissected samples the recommended quantity of 1 μg total RNA could be used in the amplification reaction. Therefore, the amplification-induced bias is probably slightly higher for microdissected samples than for macrodissected samples. Of note, one round of amplification is demonstrated to be more sensitive to low abundance transcripts than using total RNA [18-20].

A possible disadvantage of macrodissected samples is the presence of stroma cells that might disturb the epithelial tumor-specific gene expression profiles. We therefore evaluated the contribution of stroma in the macrodissected gene expression profiles. In the unsupervised clustering of twice-amplified samples, macrodissected samples clustered closer to microdissected tumor samples than to microdissected stroma samples, suggesting that epithelial tumor cells had a higher contribution to gene expression profiles than stroma cells. This observation was confirmed by linear regression analysis, indicating that the involvement of stroma in macrodissected gene expression profiles was minor. In the unsupervised clustering, samples 2 and 14 (low en high percentage of tumor epithelium, respectively) clustered relatively together with their corresponding microdissected stroma and tumor sample. Although this observation suggested an association between the surface percentage of tumor epithelium in the macrodissected sample and the degree of clustering of this sample with the microdissected tumor sample, such a clear-cut correlation could not be established. The contribution of stroma to the gene expression profiles was not strictly related to the surface percentage and was smaller than expected from the surface percentage of the stroma. However, for the sample with 15% tumor epithelium the contributions of stroma and tumor were equal, indicating that this sample contained probably too much stroma for adequate analysis of tumor-derived genes. For such samples a further enrichment for tumor epithelium is necessary and can probably be attained by macrodissection.

An explanation for our finding that the contribution of stroma is relatively small is provided by the observation that the yields of total RNA as well as of amplified mRNA from stroma samples were much lower than from equal volumes of tumor tissue. These findings are presumably due to a higher density of tumor cells and/or more transcription activity in tumor epithelium compared to stroma. Although these data are obtained by analysis of rectal carcinoma samples, our conclusions are probably applicable to other tumor types with a stroma component as well. The fact that far less mRNA is isolated from stroma than from epithelium suggests that the contribution of stroma to the overall gene expression profile will always be minor with the consequence that macrodissection might be the preferred method for other carcinoma types as well. Furthermore, in case it is absolutely necessary to discard the stroma gene expression, it might be an option to perform *in silico *microdissection [21-23]. These computational approaches have the advantage that macrodissected samples can be used, thereby leaving out the biases caused by the required second round of amplification in case of manual microdissection. However, it is important to realize that most of the *in silico *approaches are based on the assumption that tumor epithelium and stroma will equally contribute to the overall gene expression profile. In this study, we demonstrated that the stroma contribution is much smaller than expected from the surface area of the rectal carcinoma sample, which should be included in the *in silico *analysis.

Although the influence of stroma-derived RNA on the expression profiles of genes which are expressed by stroma as well as by tumor epithelium is small, expression of genes which are specific for stroma cells, might still be detectable when using macrodissection [24]. This is an additional advantage of macrodissection, because increasing evidence supports an important role for the microenvironment in carcinoma formation and progression, and therefore these stroma cells might be of great interest. For instance, expression of some stroma-specific genes appeared to be correlated with patient prognosis [2,25]. Fromique et al. [26] showed that signaling between epithelial tumor cells and fibroblasts influenced the gene expression pattern of the tumor cells. For rectal carcinoma it has been demonstrated that apart from the pathological characteristics of tumor cells the amount and type of infiltrate is also relevant for the control of cancer [27]. When tumor epithelium is selected by LMPC, this stroma-specific information is missed.

## Conclusion

Because rectal carcinoma samples contained varying amounts of stroma versus tumor epithelium, the question arose whether macrodissection could be used or whether the samples should be microdissection for gene expression profiling. Purification of tumor epithelium by laser microdissection was supposed to give the most reliable tumor-specific gene expression profiles. However, we showed that these overall gene expression profiles are affected by the required second round of mRNA amplification with random primers. The contribution of stroma to gene expression profiles of macrodissected samples was much smaller than expected on the basis of the quantified surfaces. And of even more importance, the interference of stroma cells with the overall gene expression profiles appeared to be minor. Therefore, we recommend RNA isolation of clinically resected carcinomas samples that are only enriched for tumor epithelium by macrodissection for microarray experiments.

## Methods

### Macrodissection and microdissection of tissue samples

**Figure 4:**
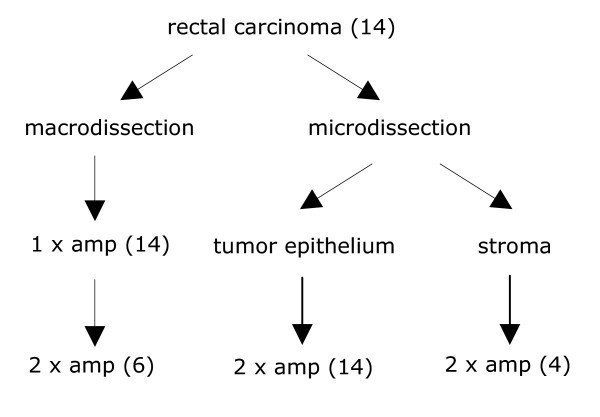
Schematic overview of the strategy used to compare macrodissection and microdissection. All 14 rectal carcinoma samples are macrodissected or microdissected for tumor epithelium, 6 macrodissected samples were in addition amplified (amp) a second round and of 4 samples the stroma was microdissected as well.

The experimental outline of this study is depicted in Figure 4. Fresh frozen rectal carcinoma samples were obtained from 14 different patients who underwent surgery in either the Leiden University Medical Center or the Leyenburg Hospital. All samples were macrodissected in a cryostat at -20°C by removing surrounding healthy tissue. Of these, two sections of 30 μm were collected for total RNA extraction. For the microdissection procedure [28], sections of 10 μm were cut and adhered to polyethylene-naphtalate (PEN) membrane slides (P.A.L.M. Microlaser Technologies AG, Bernried, Germany), followed by hydration by rinsing the slides in 100%, 75% and 50% ethanol. The samples were stained with Mayer's haematoxylin, briefly rinsed in diethylpyrocarbonate (DEPC)-treated water and dehydrated in graded ethanols. All slides were finally air-dried and stored dry at -80°C until microdissection was performed using the PALM^® ^Micro Beam microscope (P.A.L.M. Microlaser Technologies AG) for non-contact laser microdissection and pressure catapulting (LMPC). Microdissection of 0.5–1 mm^2 ^tissue took 30 to 120 minutes per sample.

Sections of 5 μm of each macrodissected sample were stained with classical haematoxylin and eosin staining and examined by light microscopy to quantify the surface percentages of tumor epithelium versus stroma. Table 1 gives an overview of the samples used and the quantified percentages of tumor epithelium.

### Total RNA extraction, mRNA amplification and labeling

For macrodissected samples, the sections were homogenized by vortexing with glass beads in RNA-Bee reagent (Tel-Test Inc., Friendswood, TX). Total RNA was extracted according to the manufacturer's protocol of RNA-Bee and purified using the Qiagen RNeasy mini kit with on-column DNase digestion according to manufacturer's instructions (Qiagen Sciences, Germantown, MD). For microdissected tissues, total RNA was isolated using the Qiagen RNeasy mini kit with on-column DNAse treatment (Qiagen). Quality of total RNAs was assessed with lab-on-a-chips on the Agilent 2100 Bioanalyzer (Agilent Technologies, Palo Alto, California). All samples were shown to be free of DNA contamination and for each sample the ratio 28S/18S was >1.5.

Amplifications were performed using Ambion's MessageAmp™ kit and protocol (Ambion Inc., Austin, TX). For macrodissected samples, of which on average 30 μg total RNA was isolated, the first amplification round was started with 1.0 μg total RNA. This first amplification round of macrodissected samples yielded on average 24 μg amplified mRNA (aRNA). The second round of amplification, using random second round primers, was started with 1.0 μg aRNA in case of macrodissected samples. For microdissected specimens the whole quantity of isolated total RNA was used (on average 30 ng total RNA) for the first round of amplification, and all aRNA was used for the second round of amplification. Yield of aRNA of these twice-amplified microdissected samples was on average 15 μg aRNA. Quality of each aRNA was checked on lab-on-a-chip (Agilent Technologies). Quantification of aRNA was performed by spectrometry at 260 nm wavelength.

Per microarray experiment, 1.0-μg aliquots of aRNA were labeled with Cy5-dUTPs (Amersham Biosciences, Buckinghamshire, UK) by direct incorporation during a reverse transcriptase reaction using the CyScribe kit, according to manufacturer's instructions (Amersham Biosciences). The labeled cDNAs were mixed with equal amounts of Cy3-dUTP-labeled cDNA from a once-amplified reference probe, consisting of equal amounts of RNA from all macrodissected samples.

### cDNA microarray

The mixture of labeled reference and sample was purified on YM30 Microcon columns (Millipore Corporation, Bedford, MA) together with 20 μg human COT-1 DNA (Invitrogen, Carlsbad, CA). After purification, 8 μg yeast tRNA (Invitrogen) and 20 μg polyadenylic acid (Sigma-Aldrich, St. Louis, MO) were added. Preheated hybridization buffer (25% formamide, 5× SSC, 0.1% SDS) was added just before hybridization at 42°C o/n in to human 18K cDNA microarrays slides, manufactured at the Central Microarray Facility (CMF) of the Netherlands Cancer Institute. Protocols, GeneID list and information about arrays are available at the website of the CMF [29].

### Data preparation

Of each slide, two images were scanned using the GeneTAC LSIV laser scanner (Genomic Solutions, Ann Arbor, MI) at different gain settings, one at which hardly any of the spots were saturated and one with a higher gain to obtain data from lowly expressed genes. Spots were quantified by using GenePix Pro 4.1 software (Axon Instruments Inc., Union City, CA). For spot selection an MS-Excel macro was used [30]. Briefly: spots were corrected for local background noise. Per dye, the intensity of each spot was normalized to the median of all spots on the array and for each spot, the ratio of the sample to the reference was calculated. Because arrays were scanned at two different settings, ratios from high gain-saturated spots were used from the low gain scans, while lowly expressed genes were used from the high gain scans. Genes saturated in both gains were rejected from analysis. For other spots, the mean of the ratios of the two scans was calculated. Finally, ratios were log_10_-transformed. Because the goal of this study was to analyze the effects of macro- and microdissection on overall gene expression profiles and not to select specific genes, only qualified genes that were present on all 45 arrays (2358 genes) were selected for further statistical analyses. The data discussed in this publication have been deposited in NCBIs Gene Expression Omnibus (GEO) [31] and are accessible through GEO Series accession number GSE2738.

### Array data analysis

Unsupervised clustering of the genes and samples was performed with Spotfire 7.2 software (Spotfire AB, Göteborg, Sweden) based on hierarchical clustering of average linkage correlation of the log_10_-transformed data. Pearson correlation coefficients and linear regression were calculated with SPSS 11.0 software for Windows (SPSS Inc., Chicago, IL). The "conservative" and "rejected" gene sets were calculated according to Nygaard et al. [9]. In summary, for the "conservative set of genes", each gene with a p-value less than 0.1 in a t-test assuming unequal variances or in a t-test assuming equal variances was removed. The "rejected set of genes" are genes which are significantly changed when two groups of samples are compared in a t-test according to the Benjamini-Hochberg procedure [32] with a false discovery rate of 1%.

## Authors' contributions

EB and SP carried out all experiments and data analysis with assistance of EL, RE and MZ. ML and TW designed the Excel macro to normalize the microarray data. CM, JK, JM, CV and LP initiated the study and supervised the data generation and analyses. PE was involved in the statistical analyses. All authors read and approved that final manuscript.
